# Supplementary material for: A Combination of Microarray-Based Profiling and Biocomputational Analysis Identified miR331-3p and hsa-let-7d-5p as Potential Biomarkers of Ulcerative Colitis Progression to Colorectal Cancer
Source: Int J Mol Sci. 2024 May 23;25(11):5699. doi: 10.3390/ijms25115699 (PMC11171846; doi:10.3390/ijms25115699)
Supplement: Supplementary file 1 [file ijms-25-05699-s001.zip › ijms-2953134-supplementary.pdf]

**Figure S1**. A total of 17 protein clusters were identified from the PPI network. In colour, the different analysed protein clusters that have been specifically described below beside the MCODE protein cluster score: 1 (**A**) score = 6.857, 2 (**B**) score = 7, 3 (**C**) score = 4, 4 (**D**) score = 4, 5 (**E**) score = 3.733, 6 (**F**) score = 3.6, 7 (**G**) score = 3.5, 8 (**H**) score = 3.455, 9 (**I**) score = 3.333, 10 (**J**), 11 (**K**) score = 3, 12 (**L**) score = 3, 13 (**M**) score = 3, 14 (**N**) score = 3.

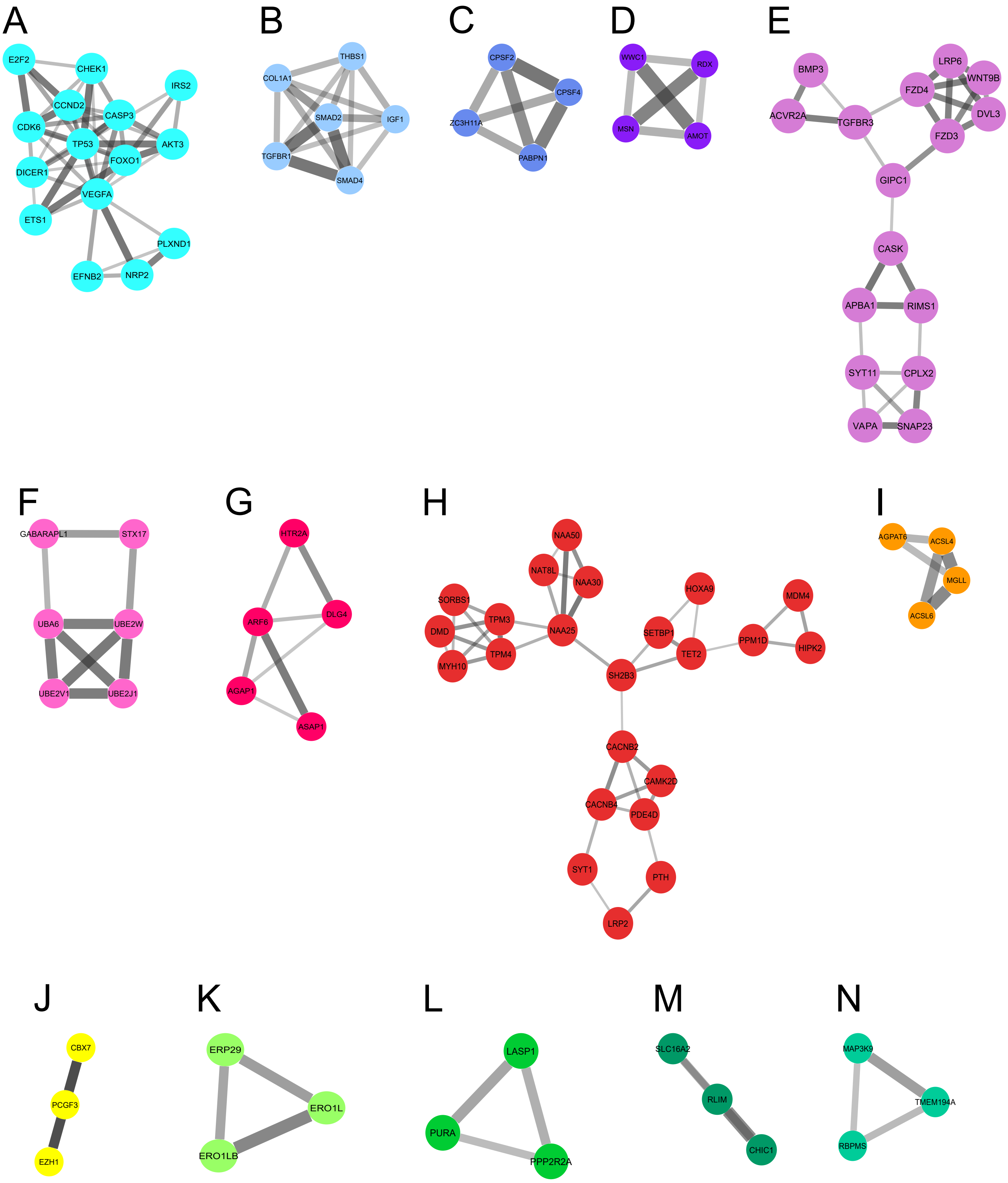

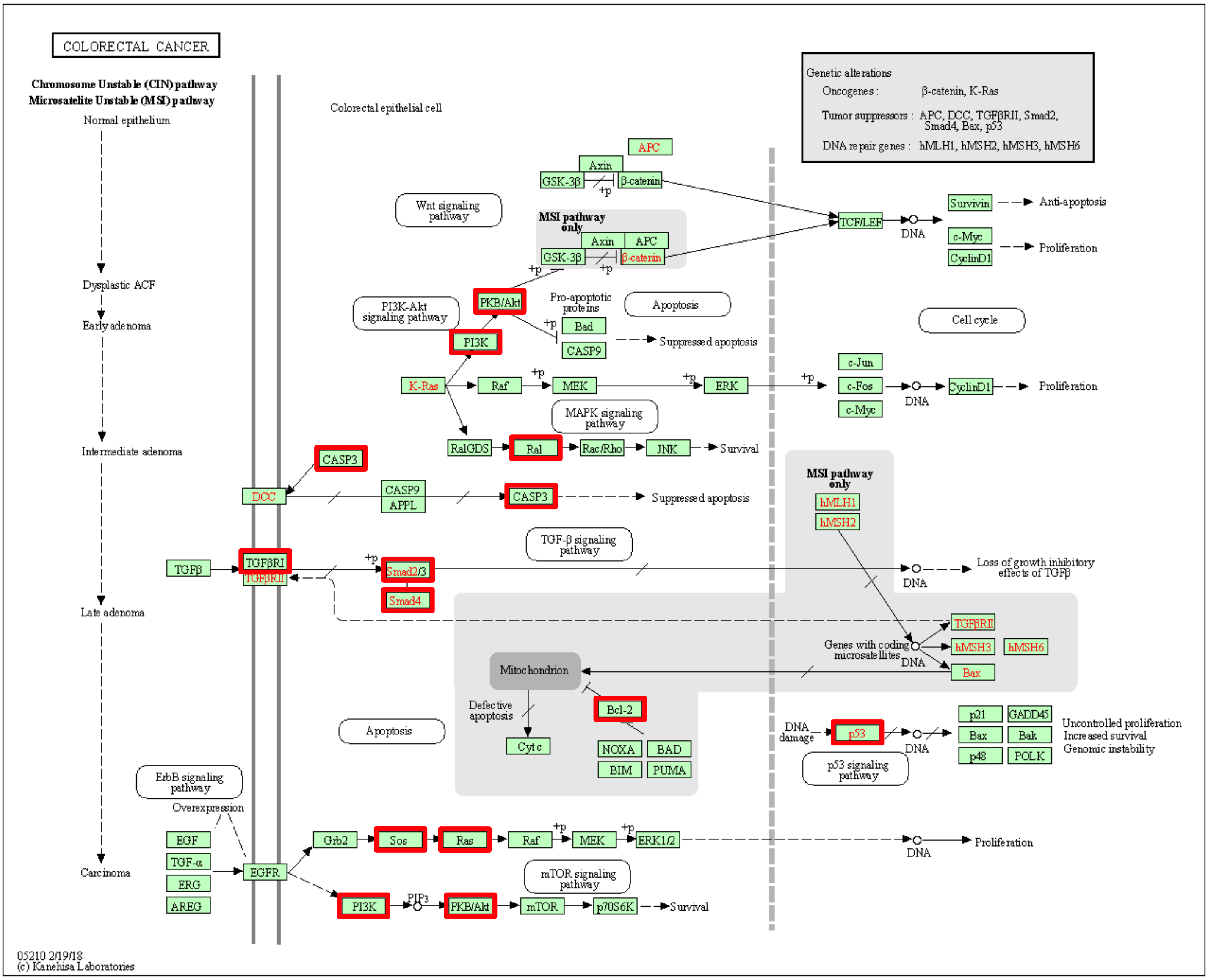


**Figure S2.** Specific genes affected in the colorectal tumoral pathway. Highlighted in red the possible proteins by our miRNA candidates. Written in red are the main oncogenes, tumoral suppressors, and DNA repair genes. Pathway map generated in DAVID web interface.

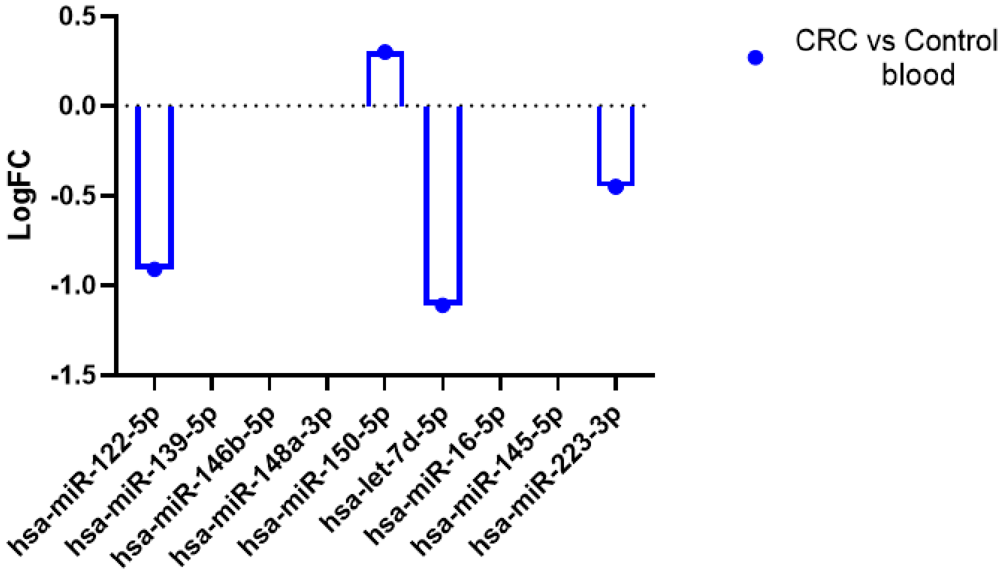


**Figure S3.** Identified circulating miRNAs in dbDEMC (database of Differentially Expressed MiRNAs in human Cancers). Hsa-miR-122-5p, hsa-let-7d-5p and hsa-miR-223-3p were downregulated, whereas hsa-miR-150-5p was upregulated in CRC compared to normal from blood samples.

| miRNA Symbol | circRNA Target Symbol | circRNA Target ID |
| --- | --- | --- |
| miR-145-5p | circ-CEP128 | circular RNA centrosomal protein 128 |
| miR-331-3p | circ-SHPRH | circular RNA SNF2 histone linker PHD RING helicase |

**Table S1.** CircRNAs inhibitors of our upregulated miRNA candidates.

**Table S2.** MRNAs targeted by our upregulated miRNA candidates.

| miRNA Symbol | mRNA Target Symbol | mRNA Target ID |
|---|---|---|
| let-7d-5p | A1CF | APOBEC1 complementation factor |
| let-7d-5p | AAK1 | AP2 associated kinase 1 |
| let-7d-5p | ABCC5 | ATP binding cassette subfamily C member 5 |
| let-7d-5p | ACSL6 | acyl-CoA synthetase long chain family member 6 |
| let-7d-5p | ACVR2A | activin A receptor type 2A |
| let-7d-5p | ADIPOR2 | adiponectin receptor 2 |
| let-7d-5p | AGAP1 | ArfGAP with GTPase domain, ankyrin repeat and PH domain 1 |
| let-7d-5p | AGO1 | argonaute RISC component 1 |
| let-7d-5p | AMER3 | APC membrane recruitment protein 3 |
| let-7d-5p | AMMECR1L | AMMECR1 like |
| let-7d-5p | AMOT | angiomotin |
| let-7d-5p | ANKRD28 | ankyrin repeat domain 28 |
| let-7d-5p | ANKRD46 | ankyrin repeat domain 46 |
| let-7d-5p | APBA1 | amyloid beta precursor protein binding family A member 1 |
| let-7d-5p | ARID3B | AT-rich interaction domain 3B |
| let-7d-5p | ARL5A | ADP ribosylation factor like GTPase 5A |
| let-7d-5p | ARMC8 | armadillo repeat containing 8 |
| let-7d-5p | ARPP19 | cAMP regulated phosphoprotein 19 |
| let-7d-5p | ASAP1 | ArfGAP with SH3 domain, ankyrin repeat and PH domain 1 |
| let-7d-5p | ATP8B4 | ATPase phospholipid transporting 8B4 (putative) |
| let-7d-5p | B4GAT1 | beta-1,4-glucuronyltransferase 1 |
| let-7d-5p | BACH1 | BTB domain and CNC homolog 1 |
| let-7d-5p | BBX | BBX high mobility group box domain containing |
| let-7d-5p | BCAP29 | B cell receptor associated protein 29 |
| let-7d-5p | BRWD3 | bromodomain and WD repeat domain containing 3 |
| let-7d-5p | BTG2 | BTG anti-proliferation factor 2 |
| let-7d-5p | BZW1 | basic leucine zipper and W2 domains 1 |
| let-7d-5p | BZW2 | basic leucine zipper and W2 domains 2 |
| let-7d-5p | C19orf47 | chromosome 19 open reading frame 47 |
| let-7d-5p | CACNA1E | calcium voltage-gated channel subunit alpha1 E |
| let-7d-5p | CACNB4 | calcium voltage-gated channel auxiliary subunit beta 4 |
| let-7d-5p | CALU | calumenin |
| let-7d-5p | CARNMT1 | carnosine N-methyltransferase 1 |
| let-7d-5p | CASP3 | caspase 3 |
| let-7d-5p | CBFA2T3 | CBFA2/RUNX1 partner transcriptional co-repressor 3 |
| let-7d-5p | CBX2 | chromobox 2 |
| let-7d-5p | CBX5 | chromobox 5 |
| let-7d-5p | CCDC141 | coiled-coil domain containing 141 |
| let-7d-5p | CCND2 | cyclin D2 |
| let-7d-5p | CCNT2 | cyclin T2 |
| let-7d-5p | CHIC1 | cysteine rich hydrophobic domain 1 |
| let-7d-5p | CLCN5 | chloride voltage-gated channel 5 |
| let-7d-5p | CNOT6L | CCR4-NOT transcription complex subunit 6 like |
| let-7d-5p | COL1A1 | collagen type I alpha 1 chain |
| let-7d-5p | COL4A1 | collagen type IV alpha 1 chain |
| let-7d-5p | CPA4 | carboxypeptidase A4 |
| let-7d-5p | CPEB1 | cytoplasmic polyadenylation element binding protein 1 |
| let-7d-5p | CPEB3 | cytoplasmic polyadenylation element binding protein 3 |
| let-7d-5p | CPED1 | cadherin like and PC-esterase domain containing 1 |
| let-7d-5p | CPSF4 | cleavage and polyadenylation specific factor 4 |
| let-7d-5p | CRY2 | cryptochrome circadian regulator 2 |
| let-7d-5p | CSRNP3 | cysteine and serine rich nuclear protein 3 |
| let-7d-5p | CYB561D1 | cytochrome b561 family member D1 |
| let-7d-5p | DDI2 | DNA damage inducible 1 homolog 2 |
| let-7d-5p | DHX57 | DExH-box helicase 57 |
| let-7d-5p | DMD | dystrophin |
| let-7d-5p | DOCK3 | dedicator of cytokinesis 3 |

| | | |
|---|---|---|
| let-7d-5p | DTX4 | deltex E3 ubiquitin ligase 4 |
| let-7d-5p | DVL3 | dishevelled segment polarity protein 3 |
| let-7d-5p | DYRK1A | dual specificity tyrosine phosphorylation regulated kinase 1A |
| let-7d-5p | DYRK2 | dual specificity tyrosine phosphorylation regulated kinase 2 |
| let-7d-5p | E2F2 | E2F transcription factor 2 |
| let-7d-5p | EDEM3 | ER degradation enhancing alpha-mannosidase like protein 3 |
| let-7d-5p | EEF2K | eukaryotic elongation factor 2 kinase |
| let-7d-5p | EIF4G2 | eukaryotic translation initiation factor 4 gamma 2 |
| let-7d-5p | ELF4 | E74 like ETS transcription factor 4 |
| let-7d-5p | ELK4 | ETS transcription factor ELK4 |
| let-7d-5p | ELOVL4 | ELOVL fatty acid elongase 4 |
| let-7d-5p | ENTPD7 | ectonucleoside triphosphate diphosphohydrolase 7 |
| let-7d-5p | EOGT | EGF domain specific O-linked N-acetylglucosamine transferase |
| let-7d-5p | ERO1A | endoplasmic reticulum oxidoreductase 1 alpha |
| let-7d-5p | ERP29 | endoplasmic reticulum protein 29 |
| let-7d-5p | FAM104A | family with sequence similarity 104 member A |
| let-7d-5p | FBXO45 | F-box protein 45 |
| let-7d-5p | FGD6 | FYVE, RhoGEF and PH domain containing 6 |
| let-7d-5p | FNDC3B | fibronectin type III domain containing 3B |
| let-7d-5p | FNIP2 | folliculin interacting protein 2 |
| let-7d-5p | FREM2 | FRAS1 related extracellular matrix 2 |
| let-7d-5p | FRMD4B | FERM domain containing 4B |
| let-7d-5p | FZD3 | frizzled class receptor 3 |
| let-7d-5p | FZD4 | frizzled class receptor 4 |
| let-7d-5p | GAS7 | growth arrest specific 7 |
| let-7d-5p | GDAP2 | ganglioside induced differentiation associated protein 2 |
| let-7d-5p | GDPD1 | glycerophosphodiester phosphodiesterase domain containing 1 |
| let-7d-5p | GIPC1 | GIPC PDZ domain containing family member 1 |
| let-7d-5p | GJC1 | gap junction protein gamma 1 |
| let-7d-5p | GPAT4 | glycerol-3-phosphate acyltransferase 4 |
| let-7d-5p | GPCPD1 | glycerophosphocholine phosphodiesterase 1 |
| let-7d-5p | GRIN3A | glutamate ionotropic receptor NMDA type subunit 3A |
| let-7d-5p | GYG2 | glycogenin 2 |
| let-7d-5p | HAS2 | hyaluronan synthase 2 |
| let-7d-5p | HECTD2 | HECT domain E3 ubiquitin protein ligase 2 |
| let-7d-5p | HIF3A | hypoxia inducible factor 3 subunit alpha |
| let-7d-5p | HMGA1 | high mobility group AT-hook 1 |
| let-7d-5p | HOOK1 | hook microtubule tethering protein 1 |
| let-7d-5p | HOXA9 | homeobox A9 |
| let-7d-5p | HTR1E | 5-hydroxytryptamine receptor 1E |
| let-7d-5p | HTR4 | 5-hydroxytryptamine receptor 4 |
| let-7d-5p | ICMT | isoprenylcysteine carboxyl methyltransferase |
| let-7d-5p | IGF2BP1 | insulin like growth factor 2 mRNA binding protein 1 |
| let-7d-5p | IKZF2 | IKAROS family zinc finger 2 |
| let-7d-5p | IL13 | interleukin 13 |
| let-7d-5p | IMPG2 | interphotoreceptor matrix proteoglycan 2 |
| let-7d-5p | IRS2 | insulin receptor substrate 2 |
| let-7d-5p | ITGB3 | integrin subunit beta 3 |
| let-7d-5p | ITGB8 | integrin subunit beta 8 |
| let-7d-5p | KATNAL1 | katanin catalytic subunit A1 like 1 |
| let-7d-5p | KCNC2 | potassium voltage-gated channel subfamily C member 2 |
| let-7d-5p | KHNYN | KH and NYN domain containing |
| let-7d-5p | KIAA0930 | KIAA0930 |
| let-7d-5p | KIF21B | kinesin family member 21B |
| let-7d-5p | LCORL | ligand dependent nuclear receptor corepressor like |
| let-7d-5p | LEPROTL1 | leptin receptor overlapping transcript like 1 |
| let-7d-5p | LINGO1 | leucine rich repeat and Ig domain containing 1 |
| let-7d-5p | LIPH | lipase H |
| let-7d-5p | LRIG2 | leucine rich repeats and immunoglobulin like domains 2 |

| | | |
|---|---|---|
| let-7d-5p | LSM11 | LSM11, U7 small nuclear RNA associated |
| let-7d-5p | LYVE1 | lymphatic vessel endothelial hyaluronan receptor 1 |
| let-7d-5p | MAP3K9 | mitogen-activated protein kinase kinase kinase 9 |
| let-7d-5p | MDM4 | MDM4 regulator of p53 |
| let-7d-5p | MED28 | mediator complex subunit 28 |
| let-7d-5p | MED8 | mediator complex subunit 8 |
| let-7d-5p | MFSD4A | major facilitator superfamily domain containing 4A |
| let-7d-5p | MGLL | monoglyceride lipase |
| let-7d-5p | MIB1 | MIB E3 ubiquitin protein ligase 1 |
| let-7d-5p | MON2 | MON2 homolog, regulator of endosome-to-Golgi trafficking |
| let-7d-5p | MSI2 | musashi RNA binding protein 2 |
| let-7d-5p | MSN | moesin |
| let-7d-5p | MTUS1 | microtubule associated scaffold protein 1 |
| let-7d-5p | MXD1 | MAX dimerization protein 1 |
| let-7d-5p | MYRIP | myosin VIIA and Rab interacting protein |
| let-7d-5p | NAA30 | N-alpha-acetyltransferase 30, NatC catalytic subunit |
| let-7d-5p | NAP1L1 | nucleosome assembly protein 1 like 1 |
| let-7d-5p | NAT8L | N-acetyltransferase 8 like |
| let-7d-5p | NEMP1 | nuclear envelope integral membrane protein 1 |
| let-7d-5p | NOL4L | nucleolar protein 4 like |
| let-7d-5p | NRAS | NRAS proto-oncogene, GTPase |
| let-7d-5p | OSMR | oncostatin M receptor |
| let-7d-5p | P2RX1 | purinergic receptor P2X 1 |
| let-7d-5p | PARD6B | par-6 family cell polarity regulator beta |
| let-7d-5p | PARM1 | prostate androgen-regulated mucin-like protein 1 |
| let-7d-5p | PARP8 | poly(ADP-ribose) polymerase family member 8 |
| let-7d-5p | PBX3 | PBX homeobox 3 |
| let-7d-5p | PCDH19 | protocadherin 19 |
| let-7d-5p | PCGF3 | polycomb group ring finger 3 |
| let-7d-5p | PDP2 | pyruvate dehyrogenase phosphatase catalytic subunit 2 |
| let-7d-5p | PDPR | pyruvate dehydrogenase phosphatase regulatory subunit |
| let-7d-5p | PEG10 | paternally expressed 10 |
| let-7d-5p | PGM2L1 | phosphoglucomutase 2 like 1 |
| let-7d-5p | PIK3IP1 | phosphoinositide-3-kinase interacting protein 1 |
| let-7d-5p | PLCXD3 | phosphatidylinositol specific phospholipase C X domain containing 3 |
| let-7d-5p | PLD5 | phospholipase D family member 5 |
| let-7d-5p | PLEKHA8 | pleckstrin homology domain containing A8 |
| let-7d-5p | POLR2D | RNA polymerase II subunit D |
| let-7d-5p | POLR3D | RNA polymerase III subunit D |
| let-7d-5p | POU2F1 | POU class 2 homeobox 1 |
| let-7d-5p | PPP1R16B | protein phosphatase 1 regulatory subunit 16B |
| let-7d-5p | PPP2R2A | protein phosphatase 2 regulatory subunit Balpha |
| let-7d-5p | PRKAR2A | protein kinase cAMP-dependent type II regulatory subunit alpha |
| let-7d-5p | PRLR | prolactin receptor |
| let-7d-5p | RALB | RAS like proto-oncogene B |
| let-7d-5p | RAMAC | RNA guanine-7 methyltransferase activating subunit |
| let-7d-5p | RBFOX1 | RNA binding fox-1 homolog 1 |
| let-7d-5p | RBMS2 | RNA binding motif single stranded interacting protein 2 |
| let-7d-5p | RDH10 | retinol dehydrogenase 10 |
| let-7d-5p | RDX | radixin |
| let-7d-5p | RICTOR | RPTOR independent companion of MTOR complex 2 |
| let-7d-5p | RNF165 | ring finger protein 165 |
| let-7d-5p | RNF170 | ring finger protein 170 |
| let-7d-5p | RNF20 | ring finger protein 20 |
| let-7d-5p | RNF217 | ring finger protein 217 |
| let-7d-5p | RNF38 | ring finger protein 38 |
| let-7d-5p | RNF44 | ring finger protein 44 |
| let-7d-5p | RPS6KA3 | ribosomal protein S6 kinase A3 |
| let-7d-5p | RRM2 | ribonucleotide reductase regulatory subunit M2 |

| | | |
|---|---|---|
| let-7d-5p | RUFY3 | RUN and FYVE domain containing 3 |
| let-7d-5p | SBNO1 | strawberry notch homolog 1 |
| let-7d-5p | SCYL3 | SCY1 like pseudokinase 3 |
| let-7d-5p | SECISBP2L | SECIS binding protein 2 like |
| let-7d-5p | SENP5 | SUMO specific peptidase 5 |
| let-7d-5p | SH2B3 | SH2B adaptor protein 3 |
| let-7d-5p | SKIL | SKI like proto-oncogene |
| let-7d-5p | SLC16A14 | solute carrier family 16 member 14 |
| let-7d-5p | SLC16A9 | solute carrier family 16 member 9 |
| let-7d-5p | SLC22A23 | solute carrier family 22 member 23 |
| let-7d-5p | SLC25A24 | solute carrier family 25 member 24 |
| let-7d-5p | SLC30A4 | solute carrier family 30 member 4 |
| let-7d-5p | SLF2 | SMC5-SMC6 complex localization factor 2 |
| let-7d-5p | SLK | STE20 like kinase |
| let-7d-5p | SMAD2 | SMAD family member 2 |
| let-7d-5p | SMCR8 | SMCR8-C9orf72 complex subunit |
| let-7d-5p | SNAP23 | synaptosome associated protein 23 |
| let-7d-5p | SNX16 | sorting nexin 16 |
| let-7d-5p | SOCS1 | suppressor of cytokine signaling 1 |
| let-7d-5p | SOCS4 | suppressor of cytokine signaling 4 |
| let-7d-5p | SOX13 | SRY-box transcription factor 13 |
| let-7d-5p | SPRYD4 | SPRY domain containing 4 |
| let-7d-5p | SSH1 | slingshot protein phosphatase 1 |
| let-7d-5p | STARD13 | StAR related lipid transfer domain containing 13 |
| let-7d-5p | STK24 | serine/threonine kinase 24 |
| let-7d-5p | STOX2 | storkhead box 2 |
| let-7d-5p | STRN | striatin |
| let-7d-5p | STX17 | syntaxin 17 |
| let-7d-5p | SURF4 | surfeit 4 |
| let-7d-5p | SYNCRIP | synaptotagmin binding cytoplasmic RNA interacting protein |
| let-7d-5p | SYT1 | synaptotagmin 1 |
| let-7d-5p | SYT11 | synaptotagmin 11 |
| let-7d-5p | TET2 | tet methylcytosine dioxygenase 2 |
| let-7d-5p | TGFBR1 | transforming growth factor beta receptor 1 |
| let-7d-5p | THBS1 | thrombospondin 1 |
| let-7d-5p | THRSP | thyroid hormone responsive |
| let-7d-5p | TMC7 | transmembrane channel like 7 |
| let-7d-5p | TMEM167A | transmembrane protein 167A |
| let-7d-5p | TMEM178B | transmembrane protein 178B |
| let-7d-5p | TMEM234 | transmembrane protein 234 |
| let-7d-5p | TMEM65 | transmembrane protein 65 |
| let-7d-5p | TMPPE | transmembrane protein with metallophosphoesterase domain |
| let-7d-5p | TNFSF9 | TNF superfamily member 9 |
| let-7d-5p | TP53 | tumor protein p53 |
| let-7d-5p | TPP1 | tripeptidyl peptidase 1 |
| let-7d-5p | TRANK1 | tetratricopeptide repeat and ankyrin repeat containing 1 |
| let-7d-5p | TRIB2 | tribbles pseudokinase 2 |
| let-7d-5p | TSPAN18 | tetraspanin 18 |
| let-7d-5p | TTL | tubulin tyrosine ligase |
| let-7d-5p | TTLL4 | tubulin tyrosine ligase like 4 |
| let-7d-5p | TUSC2 | tumor suppressor 2, mitochondrial calcium regulator |
| let-7d-5p | TXLNA | taxilin alpha |
| let-7d-5p | UGGT1 | UDP-glucose glycoprotein glucosyltransferase 1 |
| let-7d-5p | UHRF2 | ubiquitin like with PHD and ring finger domains 2 |
| let-7d-5p | USP24 | ubiquitin specific peptidase 24 |
| let-7d-5p | USP44 | ubiquitin specific peptidase 44 |
| let-7d-5p | VGLL3 | vestigial like family member 3 |
| let-7d-5p | WNT9B | Wnt family member 9B |
| let-7d-5p | XYLT1 | xylosyltransferase 1 |

| | | |
|---|---|---|
| let-7d-5p | YOD1 | YOD1 deubiquitinase |
| let-7d-5p | ZNF322 | zinc finger protein 322 |
| let-7d-5p | ZNF516 | zinc finger protein 516 |
| let-7d-5p | ZNF566 | zinc finger protein 566 |
| let-7d-5p | ZNF583 | zinc finger protein 583 |
| let-7d-5p | ZNF641 | zinc finger protein 641 |
| let-7d-5p | ZNF644 | zinc finger protein 644 |
| let-7d-5p | ZNF689 | zinc finger protein 689 |
| miR-145-5p | ABR | ABR activator of RhoGEF and GTPase |
| miR-145-5p | ABRACL | ABRA C-terminal like |
| miR-145-5p | ACVR2A | activin A receptor type 2A |
| miR-145-5p | ADD3 | adducin 3 |
| miR-145-5p | AKT3 | AKT serine/threonine kinase 3 |
| miR-145-5p | AP1G1 | adaptor related protein complex 1 subunit gamma 1 |
| miR-145-5p | AP3S1 | adaptor related protein complex 3 subunit sigma 1 |
| miR-145-5p | AREL1 | apoptosis resistant E3 ubiquitin protein ligase 1 |
| miR-145-5p | ARF6 | ADP ribosylation factor 6 |
| miR-145-5p | ARHGAP21 | Rho GTPase activating protein 21 |
| miR-145-5p | ARIH1 | ariadne RBR E3 ubiquitin protein ligase 1 |
| miR-145-5p | ARL11 | ADP ribosylation factor like GTPase 11 |
| miR-145-5p | ATP1A2 | ATPase Na+/K+ transporting subunit alpha 2 |
| miR-145-5p | ATP1B4 | ATPase Na+/K+ transporting family member beta 4 |
| miR-145-5p | ATXN7L1 | ataxin 7 like 1 |
| miR-145-5p | BBOF1 | basal body orientation factor 1 |
| miR-145-5p | BLOC1S6 | biogenesis of lysosomal organelles complex 1 subunit 6 |
| miR-145-5p | BMP3 | bone morphogenetic protein 3 |
| miR-145-5p | BTG1 | BTG anti-proliferation factor 1 |
| miR-145-5p | C5orf15 | chromosome 5 open reading frame 15 |
| miR-145-5p | CACNB2 | calcium voltage-gated channel auxiliary subunit beta 2 |
| miR-145-5p | CAMK2D | calcium/calmodulin dependent protein kinase II delta |
| miR-145-5p | CASZ1 | castor zinc finger 1 |
| miR-145-5p | CCDC25 | coiled-coil domain containing 25 |
| miR-145-5p | CCNL1 | cyclin L1 |
| miR-145-5p | CDK6 | cyclin dependent kinase 6 |
| miR-145-5p | CLCN3 | chloride voltage-gated channel 3 |
| miR-145-5p | CLIP1 | CAP-Gly domain containing linker protein 1 |
| miR-145-5p | CRKL | CRK like proto-oncogene, adaptor protein |
| miR-145-5p | DDX17 | DEAD-box helicase 17 |
| miR-145-5p | DDX46 | DEAD-box helicase 46 |
| miR-145-5p | DENND4B | DENN domain containing 4B |
| miR-145-5p | DENND5B | DENN domain containing 5B |
| miR-145-5p | DERL2 | derlin 2 |
| miR-145-5p | DLG4 | discs large MAGUK scaffold protein 4 |
| miR-145-5p | DNAL1 | dynein axonemal light chain 1 |
| miR-145-5p | DYRK1A | dual specificity tyrosine phosphorylation regulated kinase 1A |
| miR-145-5p | EFNB3 | ephrin B3 |
| miR-145-5p | EIF4EBP2 | eukaryotic translation initiation factor 4E binding protein 2 |
| miR-145-5p | ELK4 | ETS transcription factor ELK4 |
| miR-145-5p | EPB41L5 | erythrocyte membrane protein band 4.1 like 5 |
| miR-145-5p | ERG | ETS transcription factor ERG |
| miR-145-5p | ETS1 | ETS proto-oncogene 1, transcription factor |
| miR-145-5p | FAM126A | family with sequence similarity 126 member A |
| miR-145-5p | FLT1 | fms related receptor tyrosine kinase 1 |
| miR-145-5p | G3BP1 | G3BP stress granule assembly factor 1 |
| miR-145-5p | GABARAPL1 | GABA type A receptor associated protein like 1 |
| miR-145-5p | GARRE1 | granule associated Rac and RHOG effector 1 |
| miR-145-5p | GCLM | glutamate-cysteine ligase modifier subunit |
| miR-145-5p | GIT1 | GIT ArfGAP 1 |
| miR-145-5p | HIC2 | HIC ZBTB transcriptional repressor 2 |

| | | |
|---|---|---|
| miR-145-5p | HIPK2 | homeodomain interacting protein kinase 2 |
| miR-145-5p | HS6ST1 | heparan sulfate 6-O-sulfotransferase 1 |
| miR-145-5p | HTR2A | 5-hydroxytryptamine receptor 2A |
| miR-145-5p | IKZF2 | IKAROS family zinc finger 2 |
| miR-145-5p | IPO7 | importin 7 |
| miR-145-5p | JPH1 | junctophilin 1 |
| miR-145-5p | KCNN3 | potassium calcium-activated channel subfamily N member 3 |
| miR-145-5p | KLHL11 | kelch like family member 11 |
| miR-145-5p | KLHL15 | kelch like family member 15 |
| miR-145-5p | KLHL28 | kelch like family member 28 |
| miR-145-5p | LASP1 | LIM and SH3 protein 1 |
| miR-145-5p | MAPK4 | mitogen-activated protein kinase 4 |
| miR-145-5p | MMP16 | matrix metallopeptidase 16 |
| miR-145-5p | MOSMO | modulator of smoothened |
| miR-145-5p | MPLKIP | M-phase specific PLK1 interacting protein |
| miR-145-5p | MRTFB | myocardin related transcription factor B |
| miR-145-5p | MTX3 | metaxin 3 |
| miR-145-5p | MYRF | myelin regulatory factor |
| miR-145-5p | NAA25 | N-alpha-acetyltransferase 25, NatB auxiliary subunit |
| miR-145-5p | NAA50 | N-alpha-acetyltransferase 50, NatE catalytic subunit |
| miR-145-5p | NDFIP2 | Nedd4 family interacting protein 2 |
| miR-145-5p | NEDD9 | neural precursor cell expressed, developmentally down-regulated 9 |
| miR-145-5p | NFIA | nuclear factor I A |
| miR-145-5p | NSUN4 | NOP2/Sun RNA methyltransferase 4 |
| miR-145-5p | NUDT4 | nudix hydrolase 4 |
| miR-145-5p | ONECUT2 | one cut homeobox 2 |
| miR-145-5p | ORC4 | origin recognition complex subunit 4 |
| miR-145-5p | PCSK5 | proprotein convertase subtilisin/kexin type 5 |
| miR-145-5p | PLXND1 | plexin D1 |
| miR-145-5p | PODXL | podocalyxin like |
| miR-145-5p | PPP1R9A | protein phosphatase 1 regulatory subunit 9A |
| miR-145-5p | PRPSAP2 | phosphoribosyl pyrophosphate synthetase associated protein 2 |
| miR-145-5p | PURA | purine rich element binding protein A |
| miR-145-5p | RASSF2 | Ras association domain family member 2 |
| miR-145-5p | RBPMS | RNA binding protein, mRNA processing factor |
| miR-145-5p | RIMS1 | regulating synaptic membrane exocytosis 1 |
| miR-145-5p | RLIM | ring finger protein, LIM domain interacting |
| miR-145-5p | RNF216 | ring finger protein 216 |
| miR-145-5p | RREB1 | ras responsive element binding protein 1 |
| miR-145-5p | RTKN | rhotekin |
| miR-145-5p | RUNX3 | RUNX family transcription factor 3 |
| miR-145-5p | SAP30L | SAP30 like |
| miR-145-5p | SEMA6A | semaphorin 6A |
| miR-145-5p | SET | SET nuclear proto-oncogene |
| miR-145-5p | SH3BGRL2 | SH3 domain binding glutamate rich protein like 2 |
| miR-145-5p | SLC16A2 | solute carrier family 16 member 2 |
| miR-145-5p | SLC1A2 | solute carrier family 1 member 2 |
| miR-145-5p | SMAD4 | SMAD family member 4 |
| miR-145-5p | SMCR8 | SMCR8-C9orf72 complex subunit |
| miR-145-5p | SRGAP1 | SLIT-ROBO Rho GTPase activating protein 1 |
| miR-145-5p | SRGAP3 | SLIT-ROBO Rho GTPase activating protein 3 |
| miR-145-5p | SRSF10 | serine and arginine rich splicing factor 10 |
| miR-145-5p | SSBP3 | single stranded DNA binding protein 3 |
| miR-145-5p | TAGLN2 | transgelin 2 |
| miR-145-5p | TBC1D14 | TBC1 domain family member 14 |
| miR-145-5p | TBPL1 | TATA-box binding protein like 1 |
| miR-145-5p | TENT2 | terminal nucleotidyltransferase 2 |
| miR-145-5p | TMEM33 | transmembrane protein 33 |
| miR-145-5p | TPM3 | tropomyosin 3 |

| | | |
|---|---|---|
| miR-145-5p | TPM4 | tropomyosin 4 |
| miR-145-5p | TPT1 | tumor protein, translationally-controlled 1 |
| miR-145-5p | TTC14 | tetratricopeptide repeat domain 14 |
| miR-145-5p | TUG1 | taurine up-regulated 1 |
| miR-145-5p | TULP4 | TUB like protein 4 |
| miR-145-5p | UBA6 | ubiquitin like modifier activating enzyme 6 |
| miR-145-5p | UBE2W | ubiquitin conjugating enzyme E2 W |
| miR-145-5p | UBN2 | ubinuclein 2 |
| miR-145-5p | USP46 | ubiquitin specific peptidase 46 |
| miR-145-5p | VAPA | VAMP associated protein A |
| miR-145-5p | VEZF1 | vascular endothelial zinc finger 1 |
| miR-145-5p | VPS26A | VPS26, retromer complex component A |
| miR-145-5p | ZBTB20 | zinc finger and BTB domain containing 20 |
| miR-145-5p | ZBTB33 | zinc finger and BTB domain containing 33 |
| miR-145-5p | ZHX2 | zinc fingers and homeoboxes 2 |
| miR-145-5p | ZNF521 | zinc finger protein 521 |
| miR-145-5p | ZNF704 | zinc finger protein 704 |
| miR-16-5p | ABI2 | abl interactor 2 |
| miR-16-5p | ACSL4 | acyl-CoA synthetase long chain family member 4 |
| miR-16-5p | ACVR2A | activin A receptor type 2A |
| miR-16-5p | AGO1 | argonaute RISC component 1 |
| miR-16-5p | AHCYL2 | adenosylhomocysteinase like 2 |
| miR-16-5p | AK4 | adenylate kinase 4 |
| miR-16-5p | ANKIB1 | ankyrin repeat and IBR domain containing 1 |
| miR-16-5p | ANKRD33B | ankyrin repeat domain 33B |
| miR-16-5p | ANO3 | anoctamin 3 |
| miR-16-5p | AREL1 | apoptosis resistant E3 ubiquitin protein ligase 1 |
| miR-16-5p | ARL2 | ADP ribosylation factor like GTPase 2 |
| miR-16-5p | ARL3 | ADP ribosylation factor like GTPase 3 |
| miR-16-5p | ARMCX2 | armadillo repeat containing X-linked 2 |
| miR-16-5p | ARPP19 | cAMP regulated phosphoprotein 19 |
| miR-16-5p | ATP7A | ATPase copper transporting alpha |
| miR-16-5p | BACE1 | beta-secretase 1 |
| miR-16-5p | BCL2 | BCL2 apoptosis regulator |
| miR-16-5p | BTLA | B and T lymphocyte associated |
| miR-16-5p | CAPZA2 | capping actin protein of muscle Z-line subunit alpha 2 |
| miR-16-5p | CASR | calcium sensing receptor |
| miR-16-5p | CCDC6 | coiled-coil domain containing 6 |
| miR-16-5p | CCDC85C | coiled-coil domain containing 85C |
| miR-16-5p | CCND2 | cyclin D2 |
| miR-16-5p | CCNJL | cyclin J like |
| miR-16-5p | CCNT2 | cyclin T2 |
| miR-16-5p | CDC37L1 | cell division cycle 37 like 1 |
| miR-16-5p | CDV3 | CDV3 homolog |
| miR-16-5p | CHEK1 | checkpoint kinase 1 |
| miR-16-5p | CLCN5 | chloride voltage-gated channel 5 |
| miR-16-5p | CLDN2 | claudin 2 |
| miR-16-5p | CLOCK | clock circadian regulator |
| miR-16-5p | COL12A1 | collagen type XII alpha 1 chain |
| miR-16-5p | DCUN1D1 | defective in cullin neddylation 1 domain containing 1 |
| miR-16-5p | DEDD | death effector domain containing |
| miR-16-5p | DICER1 | dicer 1, ribonuclease III |
| miR-16-5p | DIXDC1 | DIX domain containing 1 |
| miR-16-5p | DMTF1 | cyclin D binding myb like transcription factor 1 |
| miR-16-5p | EFNB2 | ephrin B2 |
| miR-16-5p | ELL | elongation factor for RNA polymerase II |
| miR-16-5p | EPB41L4B | erythrocyte membrane protein band 4.1 like 4B |
| miR-16-5p | ESRRA | estrogen related receptor alpha |
| miR-16-5p | ESRRG | estrogen related receptor gamma |

| | | |
|---|---|---|
| miR-16-5p | EZH1 | enhancer of zeste 1 polycomb repressive complex 2 subunit |
| miR-16-5p | FBXL20 | F-box and leucine rich repeat protein 20 |
| miR-16-5p | FNTA | farnesyltransferase, CAAX box, alpha |
| miR-16-5p | GALNT7 | polypeptide N-acetylgalactosaminyltransferase 7 |
| miR-16-5p | GHR | growth hormone receptor |
| miR-16-5p | GPR63 | G protein-coupled receptor 63 |
| miR-16-5p | HECTD4 | HECT domain E3 ubiquitin protein ligase 4 |
| miR-16-5p | HMGA2 | high mobility group AT-hook 2 |
| miR-16-5p | HPCAL4 | hippocalcin like 4 |
| miR-16-5p | HPSE2 | heparanase 2 (inactive) |
| miR-16-5p | HTR4 | 5-hydroxytryptamine receptor 4 |
| miR-16-5p | IP6K1 | inositol hexakisphosphate kinase 1 |
| miR-16-5p | IPPK | inositol-pentakisphosphate 2-kinase |
| miR-16-5p | IRAK2 | interleukin 1 receptor associated kinase 2 |
| miR-16-5p | IST1 | IST1 factor associated with ESCRT-III |
| miR-16-5p | KIF1B | kinesin family member 1B |
| miR-16-5p | KIF3B | kinesin family member 3B |
| miR-16-5p | KIF5C | kinesin family member 5C |
| miR-16-5p | LRP2 | LDL receptor related protein 2 |
| miR-16-5p | LRP6 | LDL receptor related protein 6 |
| miR-16-5p | LUZP1 | leucine zipper protein 1 |
| miR-16-5p | MACC1 | MET transcriptional regulator MACC1 |
| miR-16-5p | MINDY2 | MINDY lysine 48 deubiquitinase 2 |
| miR-16-5p | MRAS | muscle RAS oncogene homolog |
| miR-16-5p | MTMR11 | myotubularin related protein 11 |
| miR-16-5p | MTMR3 | myotubularin related protein 3 |
| miR-16-5p | NCBP3 | nuclear cap binding subunit 3 |
| miR-16-5p | NRBP1 | nuclear receptor binding protein 1 |
| miR-16-5p | NRP2 | neuropilin 2 |
| miR-16-5p | ONECUT2 | one cut homeobox 2 |
| miR-16-5p | PDK3 | pyruvate dehydrogenase kinase 3 |
| miR-16-5p | PELI2 | pellino E3 ubiquitin protein ligase family member 2 |
| miR-16-5p | PHACTR2 | phosphatase and actin regulator 2 |
| miR-16-5p | PIP4P1 | phosphatidylinositol-4,5-bisphosphate 4-phosphatase 1 |
| miR-16-5p | POU2F1 | POU class 2 homeobox 1 |
| miR-16-5p | PPM1D | protein phosphatase, Mg2+/Mn2+ dependent 1D |
| miR-16-5p | PPP6R3 | protein phosphatase 6 regulatory subunit 3 |
| miR-16-5p | PRR15L | proline rich 15 like |
| miR-16-5p | PSAT1 | phosphoserine aminotransferase 1 |
| miR-16-5p | PTH | parathyroid hormone |
| miR-16-5p | RAB30 | RAB30, member RAS oncogene family |
| miR-16-5p | RAB9B | RAB9B, member RAS oncogene family |
| miR-16-5p | RANBP3 | RAN binding protein 3 |
| miR-16-5p | RPS6KA3 | ribosomal protein S6 kinase A3 |
| miR-16-5p | RRAGA | Ras related GTP binding A |
| miR-16-5p | RUBCNL | rubicon like autophagy enhancer |
| miR-16-5p | SALL4 | spalt like transcription factor 4 |
| miR-16-5p | SEMA3A | semaphorin 3A |
| miR-16-5p | SEMA6D | semaphorin 6D |
| miR-16-5p | SH3GL2 | SH3 domain containing GRB2 like 2, endophilin A1 |
| miR-16-5p | SIAH1 | siah E3 ubiquitin protein ligase 1 |
| miR-16-5p | SKIL | SKI like proto-oncogene |
| miR-16-5p | SLC36A1 | solute carrier family 36 member 1 |
| miR-16-5p | SLC4A4 | solute carrier family 4 member 4 |
| miR-16-5p | SLC4A7 | solute carrier family 4 member 7 |
| miR-16-5p | SLC4A8 | solute carrier family 4 member 8 |
| miR-16-5p | SLC6A4 | solute carrier family 6 member 4 |
| miR-16-5p | SLC7A2 | solute carrier family 7 member 2 |
| miR-16-5p | SLITRK6 | SLIT and NTRK like family member 6 |

| | | |
|---|---|---|
| miR-16-5p | SOS2 | SOS Ras/Rho guanine nucleotide exchange factor 2 |
| miR-16-5p | SRP72 | signal recognition particle 72 |
| miR-16-5p | SRPK1 | SRSF protein kinase 1 |
| miR-16-5p | STOX2 | storkhead box 2 |
| miR-16-5p | SUMO3 | small ubiquitin like modifier 3 |
| miR-16-5p | TAOK1 | TAO kinase 1 |
| miR-16-5p | TBL1XR1 | TBL1X receptor 1 |
| miR-16-5p | TGFBR3 | transforming growth factor beta receptor 3 |
| miR-16-5p | THUMPD1 | THUMP domain containing 1 |
| miR-16-5p | TMCC1 | transmembrane and coiled-coil domain family 1 |
| miR-16-5p | TMEM154 | transmembrane protein 154 |
| miR-16-5p | TSC22D2 | TSC22 domain family member 2 |
| miR-16-5p | TXN2 | thioredoxin 2 |
| miR-16-5p | UBAP1 | ubiquitin associated protein 1 |
| miR-16-5p | UBE2J1 | ubiquitin conjugating enzyme E2 J1 |
| miR-16-5p | UBE2V1 | ubiquitin conjugating enzyme E2 V1 |
| miR-16-5p | UBFD1 | ubiquitin family domain containing 1 |
| miR-16-5p | USP25 | ubiquitin specific peptidase 25 |
| miR-16-5p | VAPB | VAMP associated protein B and C |
| miR-16-5p | VEGFA | vascular endothelial growth factor A |
| miR-16-5p | WDR47 | WD repeat domain 47 |
| miR-16-5p | WWC1 | WW and C2 domain containing 1 |
| miR-16-5p | XPR1 | xenotropic and polytropic retrovirus receptor 1 |
| miR-16-5p | ZBTB20 | zinc finger and BTB domain containing 20 |
| miR-16-5p | ZBTB43 | zinc finger and BTB domain containing 43 |
| miR-16-5p | ZC3H11A | zinc finger CCCH-type containing 11A |
| miR-16-5p | ZDHHC14 | zinc finger DHHC-type palmitoyltransferase 14 |
| miR-16-5p | ZHX1 | zinc fingers and homeoboxes 1 |
| miR-16-5p | ZMAT3 | zinc finger matrin-type 3 |
| miR-16-5p | ZNF362 | zinc finger protein 362 |
| miR-16-5p | ZNF436 | zinc finger protein 436 |
| miR-19a-3p | AFF1 | AF4/FMR2 family member 1 |
| miR-19a-3p | ATL2 | atlastin GTPase 2 |
| miR-19a-3p | ATP2B2 | ATPase plasma membrane Ca2+ transporting 2 |
| miR-19a-3p | ATXN1L | ataxin 1 like |
| miR-19a-3p | BCL7A | BAF chromatin remodeling complex subunit BCL7A |
| miR-19a-3p | CCDC126 | coiled-coil domain containing 126 |
| miR-19a-3p | CDC42BPA | CDC42 binding protein kinase alpha |
| miR-19a-3p | CDH11 | cadherin 11 |
| miR-19a-3p | CR2 | complement C3d receptor 2 |
| miR-19a-3p | CRACD | capping protein inhibiting regulator of actin dynamics |
| miR-19a-3p | CSMD1 | CUB and Sushi multiple domains 1 |
| miR-19a-3p | DDX6 | DEAD-box helicase 6 |
| miR-19a-3p | DIPK1A | divergent protein kinase domain 1A |
| miR-19a-3p | DNAL1 | dynein axonemal light chain 1 |
| miR-19a-3p | EFNB2 | ephrin B2 |
| miR-19a-3p | EHBP1 | EH domain binding protein 1 |
| miR-19a-3p | ETV5 | ETS variant transcription factor 5 |
| miR-19a-3p | EXOC5 | exocyst complex component 5 |
| miR-19a-3p | FAM160A1 | FHF complex subunit HOOK interacting protein 1A |
| miR-19a-3p | FAM177B | family with sequence similarity 177 member B |
| miR-19a-3p | FAT3 | FAT atypical cadherin 3 |
| miR-19a-3p | FBXO28 | F-box protein 28 |
| miR-19a-3p | FOXP2 | forkhead box P2 |
| miR-19a-3p | GSKIP | GSK3B interacting protein |
| miR-19a-3p | GTF2A1 | general transcription factor IIA subunit 1 |
| miR-19a-3p | IMPDH1 | inosine monophosphate dehydrogenase 1 |
| miR-19a-3p | KCNJ2 | potassium inwardly rectifying channel subfamily J member 2 |
| miR-19a-3p | KLF10 | Kruppel like factor 10 |

| | | |
|---|---|---|
| miR-19a-3p | KLF13 | Kruppel like factor 13 |
| miR-19a-3p | MBNL2 | muscleblind like splicing regulator 2 |
| miR-19a-3p | MGAT5 | alpha-1,6-mannosylglycoprotein 6-beta-N-acetylglucosaminyltransferase |
| miR-19a-3p | NAV3 | neuron navigator 3 |
| miR-19a-3p | NF1 | neurofibromin 1 |
| miR-19a-3p | PCDH10 | protocadherin 10 |
| miR-19a-3p | PIK3CA | phosphatidylinositol-4,5-bisphosphate 3-kinase catalytic subunit alpha |
| miR-19a-3p | RPS6KA2 | ribosomal protein S6 kinase A2 |
| miR-19a-3p | SCN4B | sodium voltage-gated channel beta subunit 4 |
| miR-19a-3p | SCN8A | sodium voltage-gated channel alpha subunit 8 |
| miR-19a-3p | SCUBE3 | signal peptide, CUB domain and EGF like domain containing 3 |
| miR-19a-3p | SH3KBP1 | SH3 domain containing kinase binding protein 1 |
| miR-19a-3p | SLC2A13 | solute carrier family 2 member 13 |
| miR-19a-3p | SLC30A7 | solute carrier family 30 member 7 |
| miR-19a-3p | STEAP2 | STEAP2 metalloreductase |
| miR-19a-3p | TAF4 | TATA-box binding protein associated factor 4 |
| miR-19a-3p | TGIF1 | TGFB induced factor homeobox 1 |
| miR-19a-3p | TNRC6B | trinucleotide repeat containing adaptor 6B |
| miR-19a-3p | TNRC6C | trinucleotide repeat containing adaptor 6C |
| miR-19a-3p | TRAK2 | trafficking kinesin protein 2 |
| miR-19a-3p | TUB | TUB bipartite transcription factor |
| miR-19a-3p | USP6 | ubiquitin specific peptidase 6 |
| miR-19a-3p | ZDHHC7 | zinc finger DHHC-type palmitoyltransferase 7 |
| miR-19a-3p | ZPLD1 | zona pellucida like domain containing 1 |
| miR-19b-3p | ARFGEF1 | ADP ribosylation factor guanine nucleotide exchange factor 1 |
| miR-19b-3p | ARHGAP11A | Rho GTPase activating protein 11A |
| miR-19b-3p | ARHGEF26 | Rho guanine nucleotide exchange factor 26 |
| miR-19b-3p | ATL2 | atlastin GTPase 2 |
| miR-19b-3p | ATXN1L | ataxin 1 like |
| miR-19b-3p | BCL3 | BCL3 transcription coactivator |
| miR-19b-3p | BEND3 | BEN domain containing 3 |
| miR-19b-3p | BNC2 | basonuclin 2 |
| miR-19b-3p | BRWD1 | bromodomain and WD repeat domain containing 1 |
| miR-19b-3p | BTBD7 | BTB domain containing 7 |
| miR-19b-3p | CBLB | Cbl proto-oncogene B |
| miR-19b-3p | CCDC126 | coiled-coil domain containing 126 |
| miR-19b-3p | CDC42BPA | CDC42 binding protein kinase alpha |
| miR-19b-3p | CLOCK | clock circadian regulator |
| miR-19b-3p | CR2 | complement C3d receptor 2 |
| miR-19b-3p | CRACD | capping protein inhibiting regulator of actin dynamics |
| miR-19b-3p | CYB561D1 | cytochrome b561 family member D1 |
| miR-19b-3p | DDHD1 | DDHD domain containing 1 |
| miR-19b-3p | DIPK1A | divergent protein kinase domain 1A |
| miR-19b-3p | EMC7 | ER membrane protein complex subunit 7 |
| miR-19b-3p | EPG5 | ectopic P-granules autophagy protein 5 homolog |
| miR-19b-3p | ETV5 | ETS variant transcription factor 5 |
| miR-19b-3p | FAM76B | family with sequence similarity 76 member B |
| miR-19b-3p | FAT3 | FAT atypical cadherin 3 |
| miR-19b-3p | FBXO28 | F-box protein 28 |
| miR-19b-3p | FKBP15 | FKBP prolyl isomerase family member 15 |
| miR-19b-3p | FOXD4L1 | forkhead box D4 like 1 |
| miR-19b-3p | GFPT1 | glutamine--fructose-6-phosphate transaminase 1 |
| miR-19b-3p | GRIN2A | glutamate ionotropic receptor NMDA type subunit 2A |
| miR-19b-3p | GULP1 | GULP PTB domain containing engulfment adaptor 1 |
| miR-19b-3p | HIP1 | huntingtin interacting protein 1 |
| miR-19b-3p | IGF1 | insulin like growth factor 1 |
| miR-19b-3p | KDM6A | lysine demethylase 6A |
| miR-19b-3p | MAPK6 | mitogen-activated protein kinase 6 |
| miR-19b-3p | MBNL3 | muscleblind like splicing regulator 3 |

| | | |
|---|---|---|
| miR-19b-3p | MDFIC | MyoD family inhibitor domain containing |
| miR-19b-3p | MGAT5 | alpha-1,6-mannosylglycoprotein 6-beta-N-acetylglucosaminyltransferase |
| miR-19b-3p | NF1 | neurofibromin 1 |
| miR-19b-3p | NFATC2 | nuclear factor of activated T cells 2 |
| miR-19b-3p | NME7 | NME/NM23 family member 7 |
| miR-19b-3p | OSBPL6 | oxysterol binding protein like 6 |
| miR-19b-3p | PCDH10 | protocadherin 10 |
| miR-19b-3p | PHF20 | PHD finger protein 20 |
| miR-19b-3p | PIK3CA | phosphatidylinositol-4,5-bisphosphate 3-kinase catalytic subunit alpha |
| miR-19b-3p | PPP1R12A | protein phosphatase 1 regulatory subunit 12A |
| miR-19b-3p | RAB21 | RAB21, member RAS oncogene family |
| miR-19b-3p | RAP1A | RAP1A, member of RAS oncogene family |
| miR-19b-3p | RFX1 | regulatory factor X1 |
| miR-19b-3p | RIMKLA | ribosomal modification protein rimK like family member A |
| miR-19b-3p | RIN2 | Ras and Rab interactor 2 |
| miR-19b-3p | RORA | RAR related orphan receptor A |
| miR-19b-3p | RPS6KA2 | ribosomal protein S6 kinase A2 |
| miR-19b-3p | SCN4B | sodium voltage-gated channel beta subunit 4 |
| miR-19b-3p | SCN8A | sodium voltage-gated channel alpha subunit 8 |
| miR-19b-3p | SEC22A | SEC22 homolog A, vesicle trafficking protein |
| miR-19b-3p | SLC6A11 | solute carrier family 6 member 11 |
| miR-19b-3p | SLMAP | sarcolemma associated protein |
| miR-19b-3p | SMURF1 | SMAD specific E3 ubiquitin protein ligase 1 |
| miR-19b-3p | SUZ12 | SUZ12 polycomb repressive complex 2 subunit |
| miR-19b-3p | TM9SF3 | transmembrane 9 superfamily member 3 |
| miR-19b-3p | TNIP1 | TNFAIP3 interacting protein 1 |
| miR-19b-3p | TRAK2 | trafficking kinesin protein 2 |
| miR-19b-3p | TXK | TXK tyrosine kinase |
| miR-19b-3p | USP6 | ubiquitin specific peptidase 6 |
| miR-19b-3p | WBP4 | WW domain binding protein 4 |
| miR-19b-3p | ZBTB4 | zinc finger and BTB domain containing 4 |
| miR-19b-3p | ZDHHC7 | zinc finger DHHC-type palmitoyltransferase 7 |
| miR-19b-3p | ZMAT3 | zinc finger matrin-type 3 |
| miR-19b-3p | ZPLD1 | zona pellucida like domain containing 1 |
| miR-223-3p | ACVR2A | activin A receptor type 2A |
| miR-223-3p | CBX5 | chromobox 5 |
| miR-223-3p | CLSTN1 | calsyntenin 1 |
| miR-223-3p | ELK4 | ETS transcription factor ELK4 |
| miR-223-3p | ERO1B | endoplasmic reticulum oxidoreductase 1 beta |
| miR-223-3p | FOXO1 | forkhead box O1 |
| miR-223-3p | GTPBP8 | GTP binding protein 8 (putative) |
| miR-223-3p | HHEX | hematopoietically expressed homeobox |
| miR-223-3p | ITPR3 | inositol 1,4,5-trisphosphate receptor type 3 |
| miR-223-3p | MYH10 | myosin heavy chain 10 |
| miR-223-3p | NAA50 | N-alpha-acetyltransferase 50, NatE catalytic subunit |
| miR-223-3p | NFIA | nuclear factor I A |
| miR-223-3p | OTUD4 | OTU deubiquitinase 4 |
| miR-223-3p | PDE4D | phosphodiesterase 4D |
| miR-223-3p | PHF20L1 | PHD finger protein 20 like 1 |
| miR-223-3p | PKNOX1 | PBX/knotted 1 homeobox 1 |
| miR-223-3p | POU2F1 | POU class 2 homeobox 1 |
| miR-223-3p | PTBP2 | polypyrimidine tract binding protein 2 |
| miR-223-3p | RAB10 | RAB10, member RAS oncogene family |
| miR-223-3p | RBMS2 | RNA binding motif single stranded interacting protein 2 |
| miR-223-3p | RSBN1L | round spermatid basic protein 1 like |
| miR-223-3p | SETBP1 | SET binding protein 1 |
| miR-223-3p | SIAH1 | siah E3 ubiquitin protein ligase 1 |
| miR-223-3p | SLC24A2 | solute carrier family 24 member 2 |
| miR-223-3p | SLC8A1 | solute carrier family 8 member A1 |

| | | |
|---|---|---|
| miR-223-3p | SORBS1 | sorbin and SH3 domain containing 1 |
| miR-223-3p | TSHZ3 | teashirt zinc finger homeobox 3 |
| miR-223-3p | TUG1 | taurine up-regulated 1 |
| miR-331-3p | ARHGEF37 | Rho guanine nucleotide exchange factor 37 |
| miR-331-3p | ARRB1 | arrestin beta 1 |
| miR-331-3p | ATOH8 | atonal bHLH transcription factor 8 |
| miR-331-3p | B4GALT2 | beta-1,4-galactosyltransferase 2 |
| miR-331-3p | BCL2L2-PABPN1 | BCL2L2-PABPN1 readthrough |
| miR-331-3p | CASK | calcium/calmodulin dependent serine protein kinase |
| miR-331-3p | CBX7 | chromobox 7 |
| miR-331-3p | CCDC85C | coiled-coil domain containing 85C |
| miR-331-3p | CPLX2 | complexin 2 |
| miR-331-3p | CPSF2 | cleavage and polyadenylation specific factor 2 |
| miR-331-3p | CSNK2A1 | casein kinase 2 alpha 1 |
| miR-331-3p | DESI1 | desumoylating isopeptidase 1 |
| miR-331-3p | EFHC1 | EF-hand domain containing 1 |
| miR-331-3p | FAM126A | family with sequence similarity 126 member A |
| miR-331-3p | HDAC5 | histone deacetylase 5 |
| miR-331-3p | IGF2BP1 | insulin like growth factor 2 mRNA binding protein 1 |
| miR-331-3p | KDELR1 | KDEL endoplasmic reticulum protein retention receptor 1 |
| miR-331-3p | KSR1 | kinase suppressor of ras 1 |
| miR-331-3p | LOXHD1 | lipoxygenase homology PLAT domains 1 |
| miR-331-3p | MYRIP | myosin VIIA and Rab interacting protein |
| miR-331-3p | NACC1 | nucleus accumbens associated 1 |
| miR-331-3p | PABPN1 | poly(A) binding protein nuclear 1 |
| miR-331-3p | PTGIR | prostaglandin I2 receptor |
| miR-331-3p | RPP14 | ribonuclease P/MRP subunit p14 |
| miR-331-3p | SOCS1 | suppressor of cytokine signaling 1 |
| miR-331-3p | SPNS2 | sphingolipid transporter 2 |
| miR-331-3p | TSPAN18 | tetraspanin 18 |
| miR-331-3p | U2SURP | U2 snRNP associated SURP domain containing |
| miR-331-3p | ZBTB39 | zinc finger and BTB domain containing 39 |
| miR-331-3p | ZC3H12B | zinc finger CCCH-type containing 12B |
| miR-331-3p | ZNF609 | zinc finger protein 609 |
| miR-331-3p | ZNF652 | zinc finger protein 652 |

**Table S3.** Protein symbol of our PPI network members.

| Protein Symbol |
|---|
| AAK1 |
| ABL2 |
| ADAM19 |
| ADAM22 |
| ADCYAP1R1 |
| ADIPOR2 |
| AFF1 |
| AGFG1 |
| AGO1 |
| AGO4 |
| ALCAM |
| ANK2 |
| AP4E1 |
| ARFIP1 |
| ARL8B |
| ARPP19 |
| ARRDC3 |
| ATP11A |
| ATP2B2 |
| ATP2B4 |
| ATP6AP2 |
| ATXN1 |
| B4GALT5 |
| B4GALT6 |
| BACH2 |
| BAI2 |
| BHLHE41 |
| BMP3 |
| BMPR1A |
| BSN |
| C18orf25 |
| C18orf32 |
| C3orf58 |
| CACNA1A |
| CACNA1C |
| CALCR |
| CAPZB |
| CCDC6 |
| CCNG1 |
| CCNT2 |
| CCRN4L |
| CDC14A |
| CDKN2AIP |
| CDS1 |
| CHD7 |
| CLIC1 |
| CNTN4 |
| CS |
| CSRNP2 |

CSRNP3
CTCFL
CTDNEP1
CUL5
DCAF6
DDAH1
DDR2
DEDD
DENR
DLAT
DLG2
DOCK6
DP2
DPYSL5
DYNLL2
DYRK1A
EGR2
ELAVL4
ELOVL5
ENSA
RPL17-C18orf32
EPAS1
EPHB2
ESR1
ETNK1
EYA3
FAM126B
FAM192A
FAM63B
FAM76B
FBXO28
FBXO32
FMR1
FOSB
FOXO3
FOXP1
FOXP2
FUT8
GABRA4
GDI1
GID4
GNPDA1
GOLM1
GOSR1
GPR180
GRID2
GXYLT1
HDX
HEG1
HIF1AN

HIPK2
HNRNPU
HOXA9
IGF1
IGSF1
INO80D
ITGA11
ITGB8
KAT6A
KBTBD8
KCTD15
KDELC2
KDM2B
KIAA1549
KIF24
KIF3A
KLF6
KLF7
KMT2A
KPNA4
LAMA4
LAMC1
LCOR
LEPROTL1
LMTK2
LRP8
MAP3K4
MAP3K9
MAPK8
MARCH6
MARCH7
MASP1
MDGA1
MDM4
MET
MIPOL1
MLLT10
MMP14
MMP16
MORN4
MRFAP1
MSI2
MTMR1
NCOA1
NDRG3
NDRG4
NFASC
NFAT5
NFIA
NHS

NOL4L
NOL7
NPEPPS
NPTN
NR1D2
NR2F2
NR5A2
NRK
NSD1
OCLN
ONECUT2
P4HA2
PAFAH1B1
PAK3
PAN3
PAPD5
PAPPA
PCDH17
PDE4D
PDGFRA
PIK3CA
PLAG1
PLEKHM3
PNRC2
PPP1R11
PPP1R3B
PRKAR2A
PRKCA
PRKD3
PRKRA
PRNP
PRRT2
PTPN14
PTPRD
PTPRJ
PTPRN2
QKI
RAD23B
RALGPS2
RASSF8
RC3H1
RFX3
RICTOR
RMND5A
RORB
RTF1
SAMD8
SATB1
SBF2
SCAPER

SEPT11
SHC3
SHISA4
SIK2
SKP1
SLC25A3
SLC25A34
SLC2A3
SLC52A2
SLITRK4
SMARCD1
SOCS2
SOX11
SOX5
SOX6
SP1
SPIN1
SRSF11
SSR1
ST18
ST6GAL1
STRN
STX3
STXBP5L
SYNJ1
SYNPO2L
SYT14
SZRD1
TBC1D10B
TBC1D22B
TBL1XR1
TBX18
TCF4
TEK
TET3
TFRC
TGFA
TIA1
TMPO
TP53
TRAF6
TRIM71
TSPAN3
U2SURP
UBE2F
UBE2G1
UBN1
UGT8
USP4
USP49

VPS53
WDR47
XKR4
XPO4
YPEL3
ZBTB20
ZBTB4
ZC3H12C
ZDHHC17
ZEB1
ZFYVE26
ZNF652
ZNF704
ZNRF3

**Protein ID**

AP2 associated kinase 1
ABL proto-oncogene 2, non-receptor tyrosine kinase
ADAM metallopeptidase domain 19
ADAM metallopeptidase domain 22
ADCYAP receptor type I
adiponectin receptor 2
AF4/FMR2 family member 1
ArfGAP with FG repeats 1
argonaute RISC component 1
argonaute RISC component 4
activated leukocyte cell adhesion molecule
ankyrin 2
adaptor related protein complex 4 subunit epsilon 1
ADP ribosylation factor interacting protein 1
ADP ribosylation factor like GTPase 8B
cAMP regulated phosphoprotein 19
arrestin domain containing 3
ATPase phospholipid transporting 11A
ATPase plasma membrane Ca2+ transporting 2
ATPase plasma membrane Ca2+ transporting 4
ATPase H+ transporting accessory protein 2
ataxin 1
beta-1,4-galactosyltransferase 5
beta-1,4-galactosyltransferase 6
BTB domain and CNC homolog 2
Adhesion G protein-coupled receptor B2
basic helix-loop-helix family member e41
bone morphogenetic protein 3
bone morphogenetic protein receptor type 1A
bassoon presynaptic cytomatrix protein
chromosome 18 open reading frame 25
chromosome 18 open reading frame 32
Divergent protein kinase domain 2A,
calcium voltage-gated channel subunit alpha1 A
calcium voltage-gated channel subunit alpha1 C
calcitonin receptor
capping actin protein of muscle Z-line subunit beta
coiled-coil domain containing 6
cyclin G1
cyclin T2
Nocturnin
cell division cycle 14A
CDKN2A interacting protein
CDP-diacylglycerol synthase 1
chromodomain helicase DNA binding protein 7
chloride intracellular channel 1
contactin 4
citrate synthase
cysteine and serine rich nuclear protein 2

cysteine and serine rich nuclear protein 3
CCCTC-binding factor like
CTD nuclear envelope phosphatase 1
cullin 5
DDB1 and CUL4 associated factor 6
dimethylarginine dimethylaminohydrolase 1
discoidin domain receptor tyrosine kinase 2
death effector domain containing
density regulated re-initiation and release factor
dihydrolipoamide S-acetyltransferase
discs large MAGUK scaffold protein 2
dedicator of cytokinesis 6
Transcription factor Dp-2
dihydropyrimidinase like 5
dynein light chain LC8-type 2
dual specificity tyrosine phosphorylation regulated kinase 1A
early growth response 2
ELAV like RNA binding protein 4
ELOVL fatty acid elongase 5
endosulfine alpha
RPL17-C18orf32 readthrough
endothelial PAS domain protein 1
EPH receptor B2
estrogen receptor 1
ethanolamine kinase 1
EYA transcriptional coactivator and phosphatase 3
family with sequence similarity 126 member B
PSME3-interacting protein
Ubiquitin carboxyl-terminal hydrolase MINDY-2
family with sequence similarity 76 member B
F-box protein 28
F-box protein 32
FMRP translational regulator 1
FosB proto-oncogene, AP-1 transcription factor subunit
forkhead box O3
forkhead box P1
forkhead box P2
fucosyltransferase 8
gamma-aminobutyric acid type A receptor subunit alpha4
GDP dissociation inhibitor 1
GID complex subunit 4 homolog
glucosamine-6-phosphate deaminase 1
golgi membrane protein 1
golgi SNAP receptor complex member 1
G protein-coupled receptor 180
glutamate ionotropic receptor delta type subunit 2
glucoside xylosyltransferase 1
highly divergent homeobox
heart development protein with EGF like domains 1
hypoxia inducible factor 1 subunit alpha inhibitor

homeodomain interacting protein kinase 2
heterogeneous nuclear ribonucleoprotein U
homeobox A9
insulin like growth factor 1
immunoglobulin superfamily member 1
INO80 complex subunit D
integrin subunit alpha 11
integrin subunit beta 8
lysine acetyltransferase 6A
kelch repeat and BTB domain containing 8
potassium channel tetramerization domain containing 15
protein O-glucosyltransferase 3
lysine demethylase 2B
KIAA1549
kinesin family member 24
kinesin family member 3A
Kruppel like factor 6
Kruppel like factor 7
lysine methyltransferase 2A
karyopherin subunit alpha 4
laminin subunit alpha 4
laminin subunit gamma 1
ligand dependent nuclear receptor corepressor
leptin receptor overlapping transcript like 1
lemur tyrosine kinase 2
LDL receptor related protein 8
mitogen-activated protein kinase kinase kinase 4
mitogen-activated protein kinase kinase kinase 9
mitogen-activated protein kinase 8
E3 ubiquitin-protein ligase MARCHF6
E3 ubiquitin-protein ligase MARCHF7
MBL associated serine protease 1
MAM domain containing glycosylphosphatidylinositol anchor 1
MDM4 regulator of p53
MET proto-oncogene, receptor tyrosine kinase
mirror-image polydactyly 1
MLLT10 histone lysine methyltransferase DOT1L cofactor
matrix metallopeptidase 14
matrix metallopeptidase 16
MORN repeat containing 4
Morf4 family associated protein 1
musashi RNA binding protein 2
myotubularin related protein 1
nuclear receptor coactivator 1
NDRG family member 3
NDRG family member 4
Neurofascin
nuclear factor of activated T cells 5
nuclear factor I A
NHS actin remodeling regulator

nucleolar protein 4 like
nucleolar protein 7
aminopeptidase puromycin sensitive
neuroplastin
nuclear receptor subfamily 1 group D member 2
nuclear receptor subfamily 2 group F member 2
nuclear receptor subfamily 5 group A member 2
Nik related kinase
nuclear receptor binding SET domain protein 1
occludin
one cut homeobox 2
prolyl 4-hydroxylase subunit alpha 2
platelet activating factor acetylhydrolase 1b regulatory subunit 1
p21 (RAC1) activated kinase 3
poly(A) specific ribonuclease subunit PAN3
Terminal nucleotidyltransferase 4B
pappalysin 1
protocadherin 17
phosphodiesterase 4D
platelet derived growth factor receptor alpha
phosphatidylinositol-4,5-bisphosphate 3-kinase catalytic subunit alpha
PLAG1 zinc finger
pleckstrin homology domain containing M3
proline rich nuclear receptor coactivator 2
protein phosphatase 1 regulatory inhibitor subunit 11
protein phosphatase 1 regulatory subunit 3B
protein kinase cAMP-dependent type II regulatory subunit alpha
protein kinase C alpha
protein kinase D3
protein activator of interferon induced protein kinase EIF2AK2
prion protein
proline rich transmembrane protein 2
protein tyrosine phosphatase non-receptor type 14
protein tyrosine phosphatase receptor type D
protein tyrosine phosphatase receptor type J
protein tyrosine phosphatase receptor type N2
QKI, KH domain containing RNA binding
RAD23 homolog B, nucleotide excision repair protein
Ral GEF with PH domain and SH3 binding motif 2
Ras association domain family member 8
ring finger and CCCH-type domains 1
regulatory factor X3
RPTOR independent companion of MTOR complex 2
required for meiotic nuclear division 5 homolog A
RAR related orphan receptor B
RTF1 homolog, Paf1/RNA polymerase II complex component
sterile alpha motif domain containing 8
SATB homeobox 1
SET binding factor 2
S-phase cyclin A associated protein in the ER

Septin-11
SHC adaptor protein 3
shisa family member 4
salt inducible kinase 2
S-phase kinase associated protein 1
solute carrier family 25 member 3
solute carrier family 25 member 34
solute carrier family 2 member 3
solute carrier family 52 member 2
SLIT and NTRK like family member 4
SWI/SNF related, matrix associated, actin dependent regulator of chromatin, subfamily d, member 1
suppressor of cytokine signaling 2
SRY-box transcription factor 11
SRY-box transcription factor 5
SRY-box transcription factor 6
Sp1 transcription factor
spindlin 1
serine and arginine rich splicing factor 11
signal sequence receptor subunit 1
ST18 C2H2C-type zinc finger transcription factor
ST6 beta-galactoside alpha-2,6-sialyltransferase 1
striatin
syntaxin 3
syntaxin binding protein 5L
synaptojanin 1
synaptopodin 2 like
synaptotagmin 14
SUZ RNA binding domain containing 1
TBC1 domain family member 10B
TBC1 domain family member 22B
TBL1X receptor 1
T-box transcription factor 18
transcription factor 4
TEK receptor tyrosine kinase
tet methylcytosine dioxygenase 3
transferrin receptor
transforming growth factor alpha
TIA1 cytotoxic granule associated RNA binding protein
thymopoietin
tumor protein p53
TNF receptor associated factor 6
tripartite motif containing 71
tetraspanin 3
U2 snRNP associated SURP domain containing
ubiquitin conjugating enzyme E2 F (putative)
ubiquitin conjugating enzyme E2 G1
ubinuclein 1
UDP glycosyltransferase 8
ubiquitin specific peptidase 4
ubiquitin specific peptidase 49

VPS53 subunit of GARP complex
WD repeat domain 47
XK related 4
exportin 4
yippee like 3
zinc finger and BTB domain containing 20
zinc finger and BTB domain containing 4
zinc finger CCCH-type containing 12C
zinc finger DHHC-type palmitoyltransferase 17
zinc finger E-box binding homeobox 1
Zinc finger FYVE domain-containing protein 26
zinc finger protein 652
zinc finger protein 704
zinc and ring finger 3

**Table S4.** Common downregulated mRNAs founded in tissue samples and after targeting miRNA analysis in serum from UC and CRC patients.

| Down mRNAs in Tissue and Serum UC-CRC |
| --- |
| AHCYL2 |
| ATP1A2 |
| BNC2 |
| BTLA |
| CDV3 |
| CR2 |
| ERO1A |
| ERO1B |
| FNDC3B |
| GRIN3A |
| HHEX |
| IGF1 |
| KCNN3 |
| MDFIC |
| MXD1 |
| PPP1R16B |
| RAB30 |
| RASSF2 |
| RUNX3 |
| SETBP1 |
| SLC16A14 |
| SOCS1 |
| UBA6 |
| UBE2J1 |
